# Bioactive Carboxymethyl Starch-Based Hydrogels Decorated with CuO Nanoparticles: Antioxidant and Antimicrobial Properties and Accelerated Wound Healing In Vivo

**DOI:** 10.3390/ijms22052531

**Published:** 2021-03-03

**Authors:** Zahra Abdollahi, Ehsan Nazarzadeh Zare, Fatemeh Salimi, Iran Goudarzi, Franklin R. Tay, Pooyan Makvandi

**Affiliations:** 1 School of Chemistry, Damghan University, Damghan 36716-41167, Iran; zahra.yalda.abdollahi@gmail.com; 2 School of Biology, Damghan University, Damghan 36716-41167, Iran; f.salimi@du.ac.ir (F.S.); irangoudarzi@du.ac.ir (I.G.); 3 The Graduate School, Augusta University, Augusta, GA 30912, USA; tayfranklin7@gmail.com; 4 Istituto Italiano di Tecnologia, Centre for Materials Interface, Viale Rinaldo Piaggio 34, 56025 Pontedera, Pisa, Italy

**Keywords:** antibacterial, antioxidant, hydrogel nanocomposites, sodium carboxymethyl starch, CuO nanoparticles, wound healing

## Abstract

In this study, nanocomposite hydrogels composed of sodium carboxymethylated starch (CMS)-containing CuO nanoparticles (CMS@CuO) were synthesized and used as experimental wound healing materials. The hydrogels were fabricated by a solution-casting technique using citric acid as a crosslinking agent. They were characterized by Fourier-transform infrared spectroscopy (FTIR), energy-dispersive X-ray spectroscopy (EDS), X-ray diffraction (XRD), field emission scanning electron microscopy (FESEM), and thermogravimetric analysis (TGA) to evaluate their physicochemical properties. In addition, swelling, antibacterial activities, antioxidant activities, cytotoxicity, and in vivo wound healing were investigated to evaluate the wound healing potential of the CMS@CuO nanocomposite hydrogels. Growth inhibition of the Gram-positive and Gram-negative pathogens, antioxidant activity, and swelling were observed in the CMS@CuO nanocomposite hydrogels containing 2 wt.% and 4 wt.% CuO nanoparticles. The hydrogel containing 2 wt.% CuO nanoparticles displayed low toxicity to human fibroblasts and exhibited good biocompatibility. Wounds created in rats and treated with the CMS@2%CuO nanocomposite hydrogel healed within 13 days, whereas wounds were still present when treated for the same time-period with CMS only. The impact of antibacterial and antioxidant activities on accelerating wound healing could be ascribed to the antibacterial and antioxidant activities of the nanocomposite hydrogel. Incorporation of CuO nanoparticles in the hydrogel improved its antibacterial properties, antioxidant activity, and degree of swelling. The present nanocomposite hydrogel has the potential to be used clinically as a novel wound healing material.

## 1. Introduction

Deprivation of nutritional factors adversely affects cellular differentiation, immune functions, and collagen formation that are vital for optimal wound healing [1]. An equally important factor for appropriate wound healing is the redox state of the wound tissue [2]. The redox state is preserved via an equilibrium between oxidant and antioxidant molecules. Oxidative stress occurs in tissues and cells when there is a disbalance between the reactive oxygen species (ROS) level and the antioxidant capability of the tissue or cells to eliminate ROS and repair the damage they cause [3,4,5]. Although ROS in low levels are crucial for the commencement and progression of wound healing, high ROS levels disrupt the cellular and molecular mechanisms involved in healing. This, in turn, causes cell death and disrupts the healing process [6].

Antioxidant compounds produce encouraging results in wound healing [7,8,9]. Antioxidants are species that deactivate ROS by donating their electrons to the ROS and preventing them from receiving electrons from vital molecules such as deoxyribonucleic acids (DNAs), proteins, and lipids [10]. Damage occurs when the level of antioxidant molecules is inadequate to protect the cells and tissue from oxidative stress [11]. Antioxidants act via enzymatic or nonenzymatic reactions that occur intracellularly or within organelles [12]. Chronic wounds in the human body possess high levels of oxidative stress [12]. Treatment of chronic wounds is often accompanied by local administration of antioxidants and antimicrobial agents to prevent infection of the wound site.

Natural polymers have attracted attention for wound treatment because of their biodegradability, biocompatibility, and non-toxic nature [13,14]. Starch is a low-cost, biodegradable natural polymer. Because of its low gel content, starch is incapable of forming a hydrogel without additional chemical and/or physical modification [15]. Carboxymethylation is a common method to improve the physical and mechanical properties of starch. Carboxymethylated starch (CMS) is a water-soluble starch derivative with a large number of carboxymethyl groups on the starch backbone [16]. This water-soluble starch version has been used extensively in the food industry [17], as well as for textile printing [18], drug delivery [19], and wound dressings [20].

The incorporation of metal oxide nanoparticles into natural polymers is often performed to improve the physicochemical, mechanical, and biological properties of polymers. Metal oxide nanoparticles possess outstanding properties in the regeneration of collagen. Many nanoparticles also possess antimicrobial and antioxidant properties [21]. Copper oxide (CuO) nanoparticles have been used extensively in biomedical applications such as anti-cancer therapy [22], drug delivery [23], and wound healing [24] because of their biocompatibility, low toxicity, and antimicrobial properties [25].

Biologically-based nanocomposite hydrogels are valuable alternatives to commercially available synthetic wound healing materials. Antibacterial and antioxidative bio-based nanocomposite hydrogels that can accelerate the wound healing process are in heavy demand for soft tissue engineering applications. Previous studies have reported the use of hydrogels and nanocomposites films in wound healing applications [21,26,27,28,29,30].

**Figure 1:**
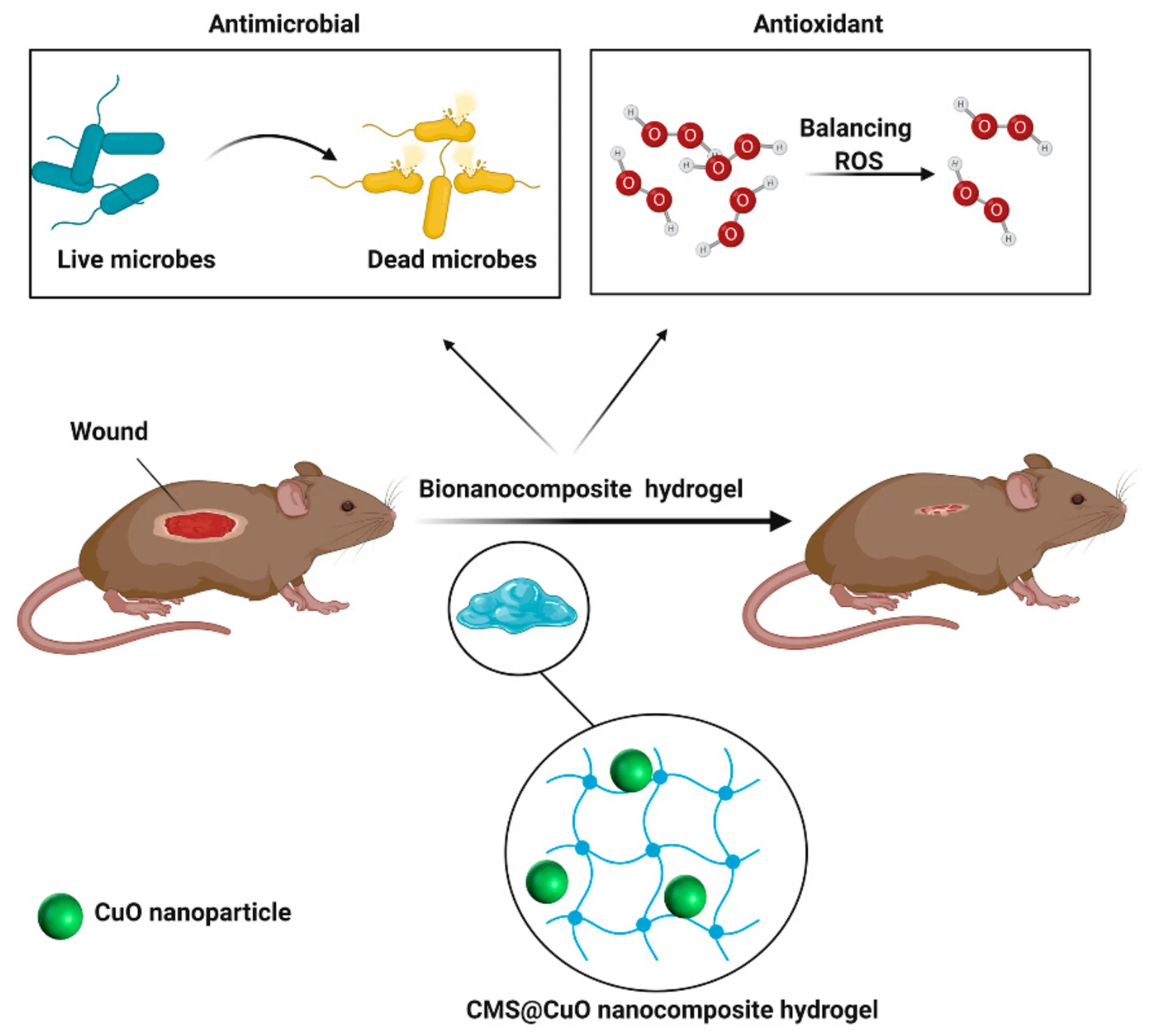
Schematic of the application of bioactive carboxymethylated starch (CMS)-containing CuO nanoparticles (CMS@CuO) nanocomposite hydrogel in wound healing. ROS: reactive oxygen species.

In the present study, new nanocomposite hydrogels were designed to possess antimicrobial and antioxidant properties. Nanocomposite hydrogels consisted of sodium CMS as a natural polymer and CuO nanoparticles for the enhancement of in vivo wound healing (Figure 1). Different versions of the nanocomposite hydrogel were fabricated using the solution-casting technique and varying the concentrations of the CuO nanoparticles. The hydrogels were characterized by Fourier transform infrared (FTIR), energy-dispersive X-ray spectroscopy (EDS), X-ray diffraction (XRD), field emission scanning electron microscopy (FESEM), and thermogravimetric analysis (TGA). The antioxidant activity of different versions of the nanocomposite hydrogel was evaluated by the 2,2-diphenyl-1-picryl-hydrazyl-hydrate (DPPH) free radical assay. The antibacterial activities, cytotoxicity, and in vivo wound healing potential were also examined. The null hypothesis to be tested was that there was no difference in the antioxidant activity, antimicrobial activity, and in vivo wound healing potential between hydrogels prepared from CMS incorporating CuO nanoparticles (CMS@CuO) and those prepared with CMS only.

## 2. Results

### 2.1. Characterization of Nanocomposite Hydrogel

**Figure 2:**
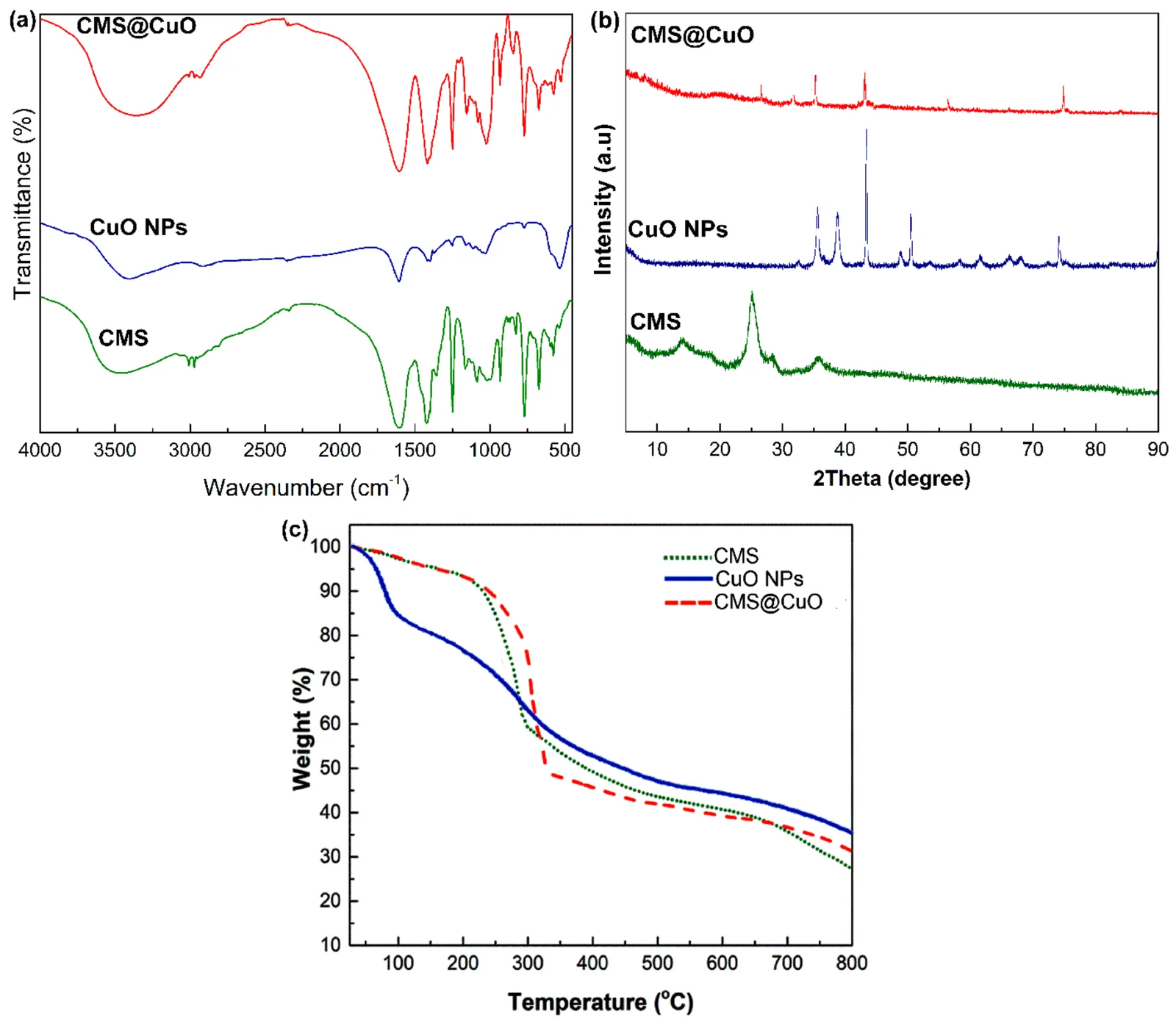
Fourier transform infrared (FTIR) (**a**), X-ray diffraction (XRD) (**b**), and thermogravimetric analysis (TGA) (**c**) of CMS, CuO nanoparticles, and the CMS@2%CuO nanocomposite hydrogel.

The infrared spectra of CMS, CuO nanoparticles, and CMS@2%CuO nanocomposite hydrogel are shown in Figure 2a. In the FTIR spectrum of CMS, the broad peak around 3500 cm^−1^ was attributed to the stretching vibrations of OH. Two peaks at 1601 cm^−1^ and 1420 cm^−1^ were associated with unsymmetrical and symmetrical stretching vibrations of the COO^−^, respectively. This result is in agreement with the literature [31,32]. In the spectrum of the CuO nanoparticles, two significant peaks that appeared at around 500 cm^−1^ and 605 cm^−1^ were attributed to the stretching vibration of Cu–O [33,34]. The peak around 1063 cm^−1^ was associated with the vibration of OH, which indicates the presence of a large number of OH groups [33,34]. A wide peak appeared in the 3200–3500 cm^−1^ was ascribed to the stretching vibration of the O–H group’s surface [33,34]. The sharp peak that appeared at around 1610 cm^−1^ was attributed to the stretching vibration of H-OH [34]. In the spectrum of CMS@2%CuO, similar peaks related to CMS and CuO nanoparticles were observed, with slightly differences in intensity and peak positions because of the interactions between hydroxyl groups from the CuO nanoparticle surface and the CMS biopolymer matrix.

X-ray diffraction patterns of CMS, CuO nanoparticles, and CMS@2%CuO nanocomposite hydrogel are shown in Figure 2b. The pattern of CMS was amorphous, when compared to the XRD pattern of starch (not shown). The reduction in crystallinity may be ascribed to the replacement of the OH groups by the COO^−^ groups [16,35]. The result suggests that increase in the degree of substitution reduces crystallinity [16,35]. The pattern of CuO nanoparticles showed sharp peaks at 33 °, 35 °, 40 °, 43 °, 48 °, 50 °, 60 °, 65 °, 68 °, and 75 °. These peaks were related to (110), (002), (111), (112), (020), (202), (113), (310), (220), and (004) planes of CuO [36]. The sharp peaks that appeared in the diffraction pattern of CuO nanoparticles indicate that the synthesized CuO nanoparticles are highly crystalline with a monoclinic structure [36]. The XRD pattern of the CMS@2%CuO nanocomposite hydrogel showed a semicrystalline nature. The peak at 31° was ascribed to residual citric acid from esterification [37]. The low-intensity peaks that appeared at 35°, 43°, and 75° suggested that CuO nanoparticles were incorporated into CMS.

The thermal stability of the CuO nanoparticles, CMS, and CMS@2%CuO nanocomposite hydrogel was evaluated at a temperature range of 25–800 °C in an inert atmosphere. The TGA curves of the CuO nanoparticles, CMS, and CMS@2%CuO nanocomposite hydrogel are shown in Figure 2c. Two major weight losses were observed on the TGA curve of CMS [38]. The first weight loss (50–150 °C) and second weight loss (250–650 °C) were attributed to water evaporation and CMS decomposition, respectively. Carbonization and ash formation occurred at high temperatures (up to 650 °C) [38]. It has been reported that carboxymethylation reduced the thermal stability of starch materials [39]. In the TGA curve of CuO nanoparticles, ~22% weight loss was seen in the range of 30–200 °C, which was attributed to water evaporation. Approximately 40% weight loss was seen from 200 °C to 800 °C, which was attributed to the organic matter decomposition of the specimen [40]. The CMS@2%CuO nanocomposite hydrogel was thermally more stable than CMS. These results indicate that the incorporation of CuO nanoparticles into CMS increased the thermal stability of the nanocomposite hydrogel. In addition, the residual weight considerably increased after the incorporation of CuO nanoparticles into CMS. The residue weight of the CuO nanoparticles, CMS, and CMS@2%CuO nanocomposite hydrogel at 800 °C was around 40%, 29%, and 35%, respectively. The effect of a larger amount of CuO nanoparticles in FT-IR spectrum, XRD pattern, EDS spectrum, and TGA curve is shown in Figure S1 (Supplementary data).

**Figure 3:**
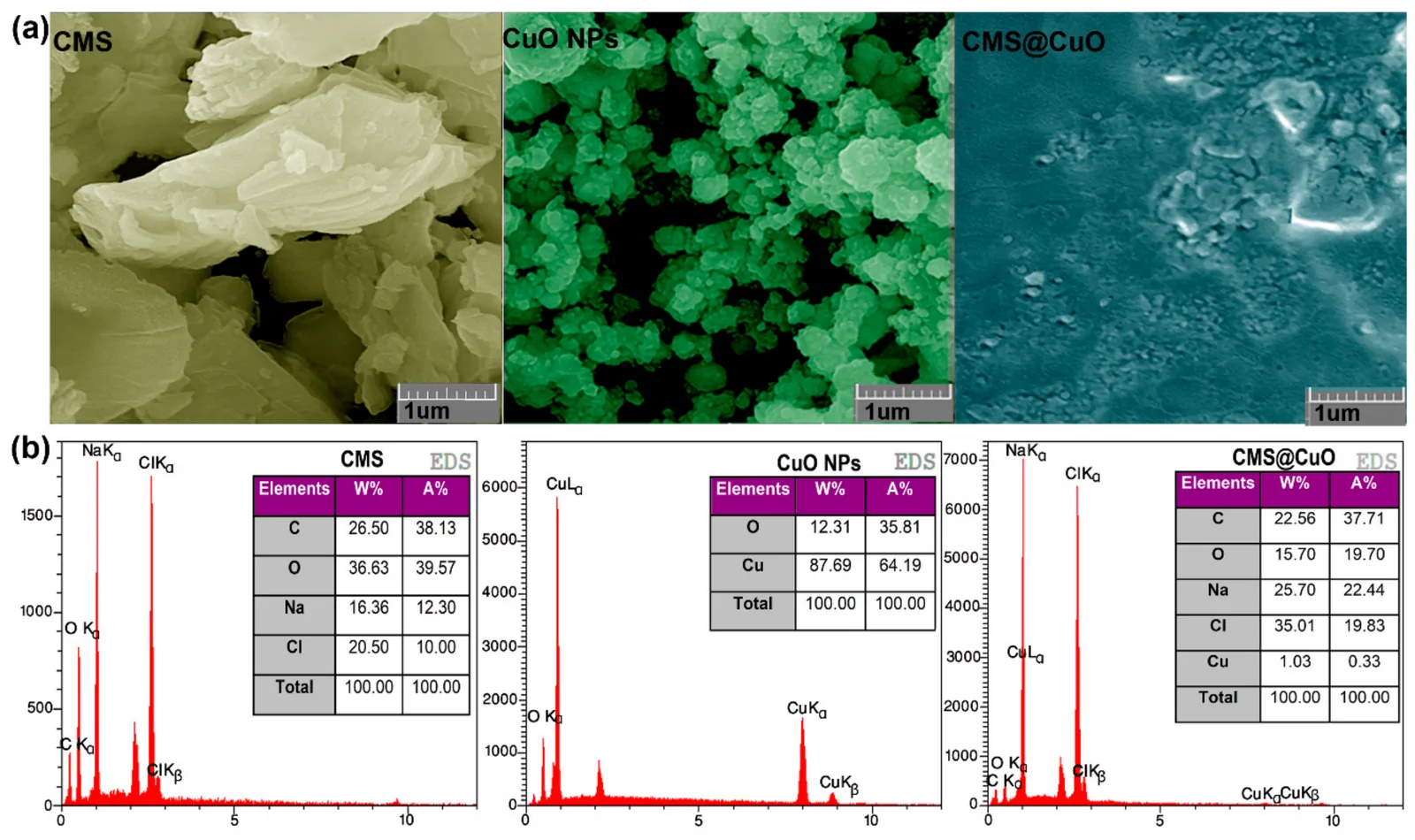
Field emission scanning electron microscopy (FESEM) images (**a**) and energy-dispersive X-ray spectroscopy (EDS) spectra and tabulated data (**b**) of CMS, CuO nanoparticles, and the CMS@2%CuO nanocomposite hydrogel. CMS: carboxymethylated starch; NPs: nanoparticles.

Field emission scanning electron microscopy was used to examine the shape and size of the prepared materials (Figure 3a). The CMS appeared microscopically as irregular granules. The CuO nanoparticles were predominantly spherical with particle sizes between 30–50 nm in diameter [35,41]. The surface of the CMS@2%CuO nanocomposite hydrogel appeared amorphous, with embedded agglomerates of CuO nanoparticles within the CMS matrix. Because of their high surface-to-volume ratio, nanoparticles tend to agglomerate to minimize their surface energy [42]. Agglomeration of the CuO nanoparticles accounts for the surface roughness of the nanocomposite hydrogel. This may be explained by the creation of a complex between the CuO nanoparticles and the COO^−^ functionalities present on the CMS backbone. The surface roughness of the hydrogel is conducive to cell growth and proliferation by supporting cell attachment and secretion of the extracellular matrix [21].

The chemical composition of CMS, CuO nanoparticles, and the CMS@2%CuO nanocomposite hydrogel was evaluated with EDS (Figure 3b). The existence of Na and Cl in the CMS specimens indicates that the biopolymer was successfully prepared. The presence of Cu in the CMS@2%CuO nanocomposite hydrogel indicates that CuO nanoparticles were successfully incorporated into the hybrid hydrogel.

### 2.2. Swelling Study

**Figure 4:**
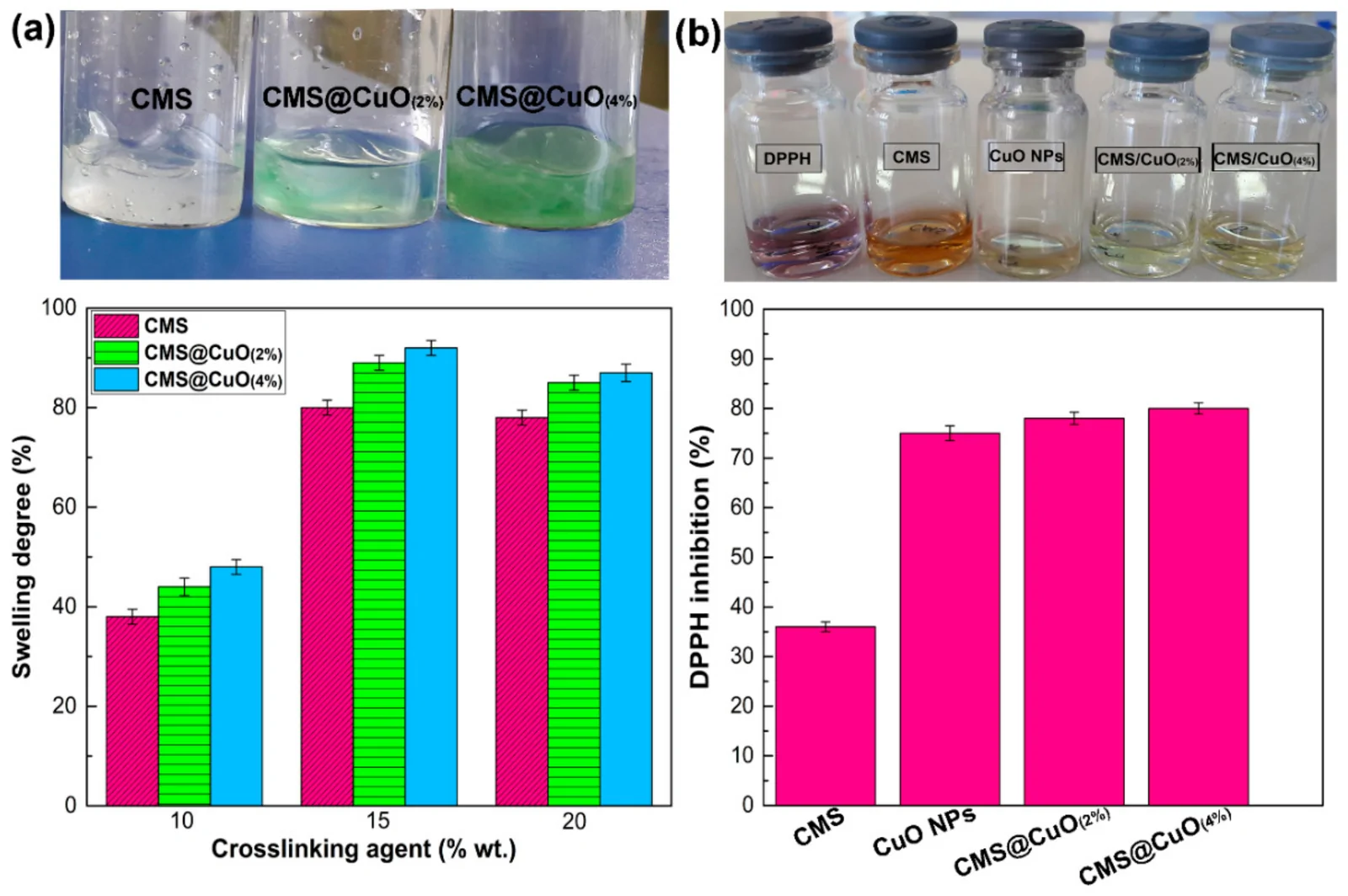
Histograms and photographs depicting the swelling behavior of the CMS hydrogel and the CMS@CuO nanocomposite hydrogels containing 2 wt.% and 4 wt.% CuO nanoparticles. The histogram on the left shows swelling in the presence of different concentrations of citric acid as crosslinking agent (**a**). The histogram on the right and the photograph above show the antioxidant activity of CMS, CuO nanoparticles and the CMS@CuO nanocomposite hydrogels with 2 wt.% and 4 wt.% CuO nanoparticles (**b**).

The degree of swelling provides an indication of the extent of crosslinking of a hydrogel network. Figure 4a shows the degree of swelling of CMS, the CMS@2%CuO nanocomposite hydrogel, and the CMS@4%CuO nanocomposite hydrogel with a slow increase of the citric acid concentration within a 60 min period. At 15 wt.% citric acid concentration, CMS had a swelling degree of ~82%. There was a drastic reduction of the degree of swelling at a high concentration of citric acid (i.e., 20 wt.%). This was attributed to the creation of covalent bonds bridging the functional groups of the CMS chains and increasingly rigid hydrogel network. When CuO nanoparticles were added to the CMS matrix, the degree of swelling was higher than what was observed for the CMS hydrogel. This may be explained by the hydrophilic nature of the CuO nanoparticles. A higher degree of swelling of the CMS matrix enables faster absorption of wound exudates. This, in turn, keeps the wound dry and inhibit airborne infection. The degree of swelling of the hydrogel matrix was further augmented when the concentration of CuO nanoparticles increased from 2 wt.% to 4 wt.% in the nanocomposite hydrogel. These results indicate that CuO nanoparticles improve the swelling property of the CMS network in a similar manner as increasing citric acid concentration.

### 2.3. Antioxidant Study

Skin is the primary barrier separating the body from the outside environment. Impaired wound healing is dangerous to the body. Delayed wound healing is observed in conditions such as old age and defects of the immune system [43].

In the inflammation phase of wound healing, leucocytes, neutrophils, and monocytes are attracted to the wound site. These immune cells secrete metabolites such as proteolytic enzymes, pro-inflammatory cytokines, and a large amount of ROS to protect the body against invading pathogens. Consequently, a low level of ROS is essential for optimal wound healing. However, high ROS concentration is detrimental because of the reactivity of these species, which adversely affect cellular mechanism. Healing of wounds or inflamed tissues under a respiratory burst condition is difficult because ROS such as hydroxyl radicals damage epithelial cells, proteins, and DNA. Previous studies have shown that the levels of antioxidants such as vitamin E, ascorbate, and glutathione are reduced (60–70%) in inflamed tissues when compared with normal skin. This reduction is further aggravated in the aged, diabetics, and subjects with immunosuppression [44]. Many studies have shown, using in vitro assays such as the DPPH assay, that the application of exogenous and endogenous antioxidants is of value in accelerating wound healing [27,45,46,47].

Accordingly, the antioxidant potential of the nanocomposite hydrogel was evaluated using the DPPH assay. Figure 4b shows the antioxidant activity of CMS, CuO nanoparticles, the CMS@2%CuO nanocomposite hydrogel, and the CMS@4%CuO nanocomposite hydrogel. Data of antioxidant activity of tested samples are shown in Table S1 (Supplementary data). The color of the DPPH solution gradually changed from dark violet to light yellow, in the order CMS@4%CuO > CMS@2%CuO > CuO nanoparticles > CMS. This color change is reflective of the potent antioxidant properties of the materials used in the present study. The antioxidant activity of a material is related to its capability to provide an active hydrogen atom or transfer electron. Consequently, the chemical structure of the material plays a significant role in its antioxidant activity [48]. The antioxidant activity of CMS, CuO nanoparticles, CMS@2%CuO, and CMS@4%CuO after 60 min in a DPPH solution was 37%, 75%, 80%, and 82%, respectively (Figure 4b). The carboxylate group in the pyran rings of the CMS plays a vital role in antioxidant activity [48,49]. This result is in agreement with the antioxidant activity of polysaccharides such as sodium alginate and gum tragacanth, which contain carboxylate in their structures [48,49]. The CuO nanoparticles also demonstrated antioxidant activity because of the hydroxyl groups present on their surface. The antioxidant activity of CuO nanoparticles may be attributed to the transfer of electrons located on the oxygen atom to the odd electron located at the nitrogen atom in DPPH, thereby causing a decrease in absorbance at 517 nm [50]. Increasing the concentration of CuO nanoparticles from 2 wt.% to 4 wt.% resulted in augmentation of the antioxidant activity of the nanocomposite hydrogel. These hybrid hydrogels may be used for relief of oxidative stress at a wound site.

### 2.4. Antibacterial Study

**Figure 5:**
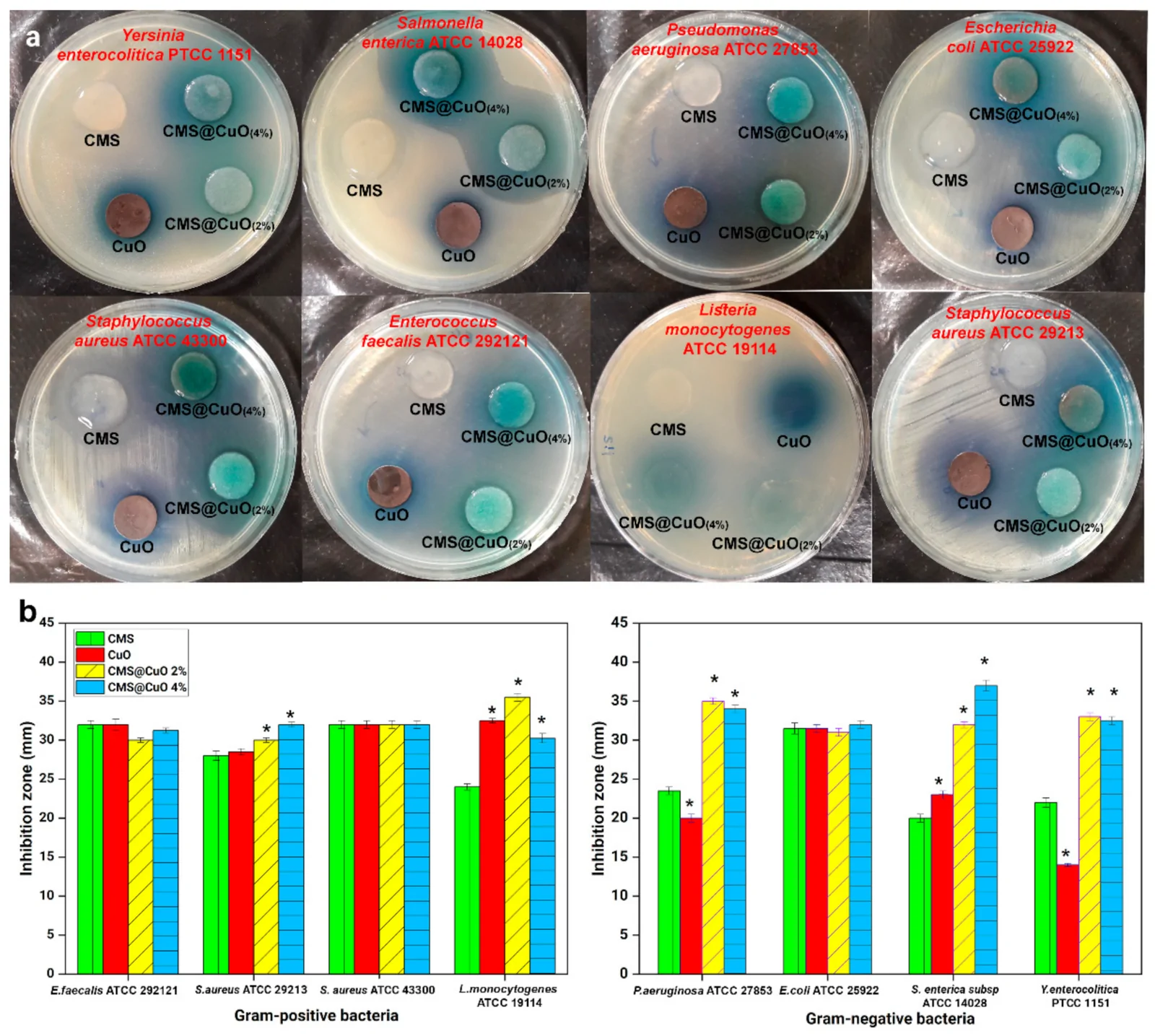
Antibacterial activity of CMS, CuO nanoparticles, the CMS@2%CuO nanocomposite hydrogel, and the CMS@4%CuO nanocomposite hydrogel against different Gram-positive and Gram-negative bacteria. (**a**) cell cultures (**b**) charts. For each chart, columns marked with asterisks (*) denote significant difference compared with pure CMS (*p* < 0.05).

The antibacterial potential of CMS, CuO nanoparticles, and the nanocomposite hydrogels with 2 wt.% or 4 wt.% CuO nanoparticles are reflected by the size of the inhibition zones against eight pathogenic bacteria in Figure 5. For the CMS, antibacterial activities against Gram-positive and Gram-negative bacteria were observed after 24 h of incubation with the diameter of inhibition zone between 20 mm and 32 mm. Although pure starch does not have antibacterial activities [51], the presence of a carboxylate group in the CMS structure is probably responsible for its antibacterial activities. Copper oxide nanoparticles also exhibited antibacterial activities against the eight bacterial species, with the diameter of inhibition zones between 20 mm and 32 mm [52,53]. The antibacterial activities of the CuO nanoparticles are attributed to ROS generation by the released metal ions, which cause malfunction of the bacteria cell membranes [53].

Incorporation of CuO nanoparticles into CMS significantly increased antibacterial activity against *P. aeruginosa*, *S. aureus*, *S. enterica*, *Y. enterocolitica*, and *L. monocytogenes* (in presence of 2 wt.% and 4 wt.% CuO nanoparticles) (*p* < 0.05). The synergistic effect was higher for *P. aeruginosa*, *Y. eterocolitica,* and *L. monocytogenes* in the CMS@2%CuO than the CMS@4%CuO nanocomposites hydrogels. Taken together, the results indicate that the inclusion of antibacterial components (CMS and CuO nanoparticles) improves the antibacterial function of the hydrogel destined for wound healing.

### 2.5. In Vitro Cell Cytotoxicity

**Figure 6:**
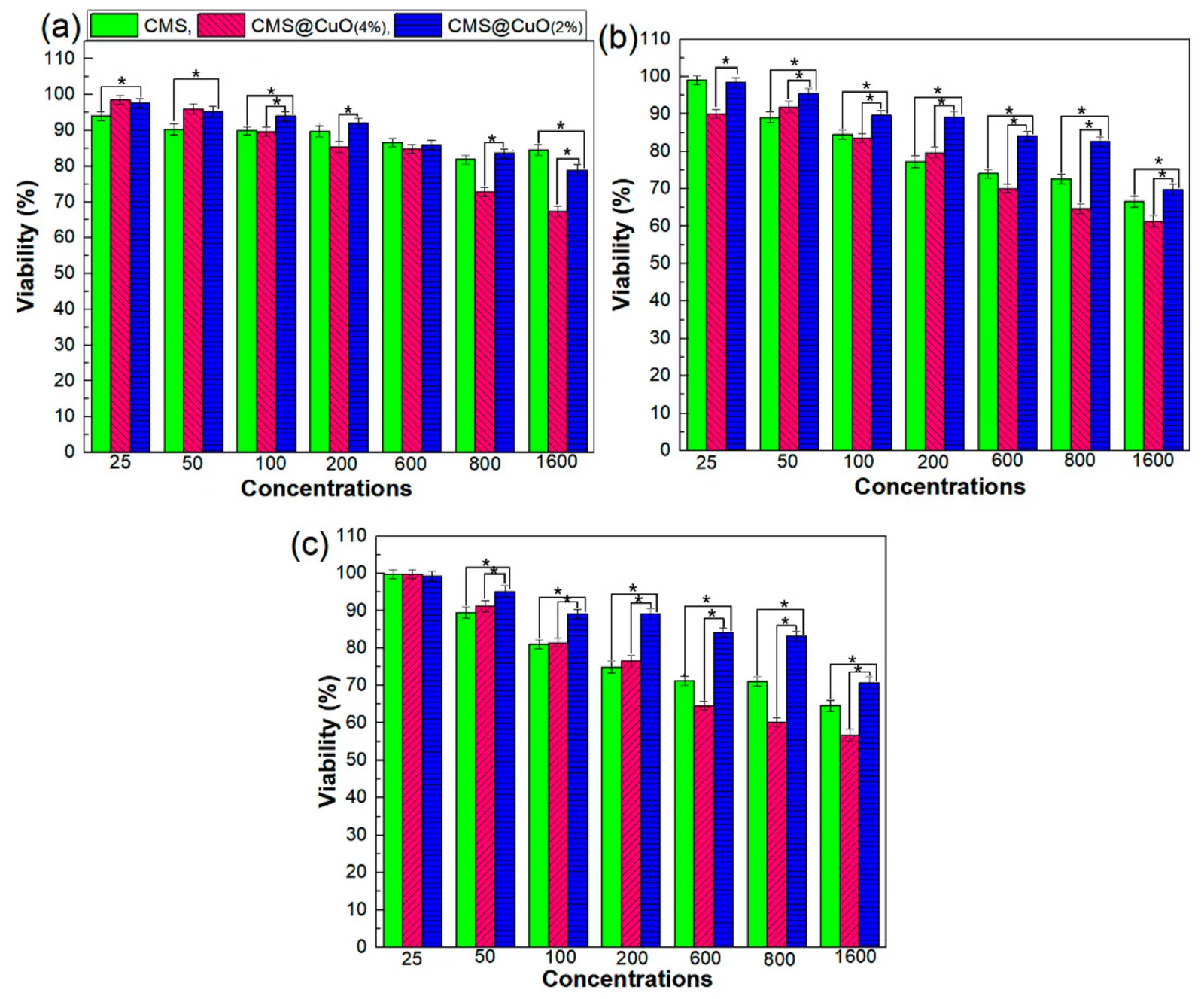
Cell viability (%) of human fibroblasts after exposure to CMS, CMS@2%CuO, and CMS@4%CuO specimens in the culture medium for 24 h (**a**), 48 h (**b**), and 72 h (**c**). Asterisks (*) indicate a significant difference (*p* < 0.05) between viability percent of treated cells with CMS@2%CuO as most efficient hydrogel and ones treated with CMS and CMS@4%CuO in one-way ANOVA test at a confidence interval of 95%.

Cytotoxicity testing utilizes tissue cells in vitro to detect cell growth, proliferation, and morphological changes. Figure 6 represents the percentage cell viability of human fibroblasts that were cultured in presence of different concentrations of CMS, CMS@2%CuO, and CMS@4%CuO specimens (25–1600 µg mL^−1^) after 24 h, 48 h, and 72 h. There was no significant difference in the viability of the fibroblasts after exposure to CMS at all concentrations examined after 24 h of incubation. The viability of CMS@CuO nanocomposite hydrogels was dose-dependent for all tested time periods. Cell viability decreased with increasing CuO nanoparticle concentration within the CMS@CuO nanocomposite hydrogels. Incorporation of 4 wt.% CuO nanoparticles into the CMS resulted in significantly more severe toxicity when compared with CMS and CMS@2%CuO after 24, 48, and 72 h of incubation. The effect of increasing CuO nanoparticle concentration on cytotoxicity has previously been reported [54]. The cytotoxicity of CuO nanoparticles is probably caused by an increase in ROS production, genotoxicity, and an increase in multi-xenobiotic resistance transport activity [53,55].

### 2.6. In Vivo Wound Healing Study

**Figure 7:**
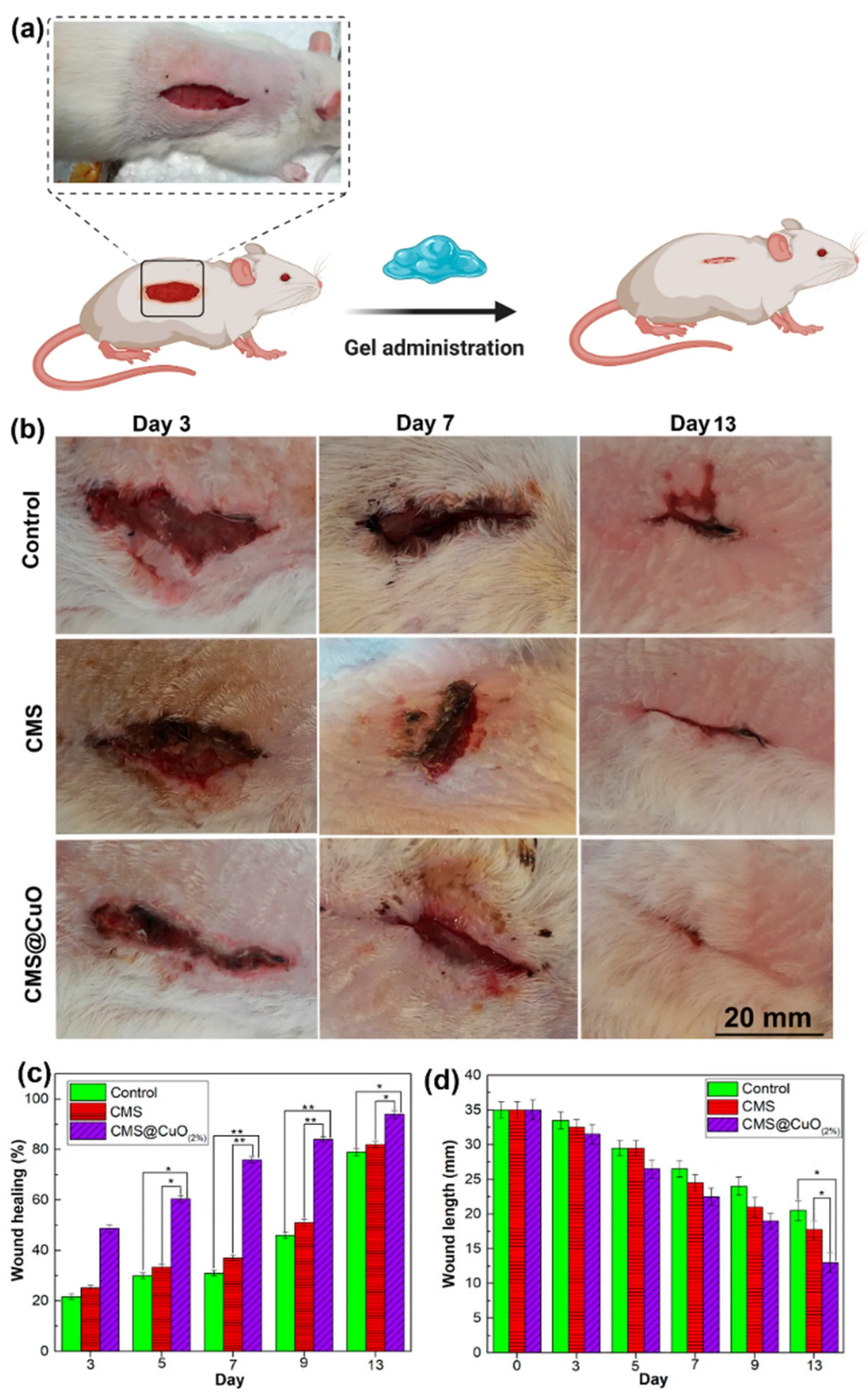
Macroscopic photographs of the wounds treated with the control, CMS, and the CMS@2%CuO nanocomposite hydrogel specimens at different time periods (**a**,**b**) and histograms of the percentage of wound healing (**c**) and wound length (**d**). For each chart, columns labeled with an asterisk (*^,^**) are significantly different from the control (*p* < 0.05 and *p* < 0.01).

Figure 7 shows the results of in vivo wound healing in a rat model. The extent of wound healing was significantly higher on day 5 (*p* < 0.05), day 7 (*p* < 0.01), day 9 (*p* < 0.01), and day 13 (*p* < 0.05) in the CMS@2%CuO nanocomposite hydrogel-treated wounds, compared with those treated with the CMS hydrogel or the control group. A significant difference was observed in wound healing percentage between wounds treated with CMS and those treated with CMS@2%CuO hydrogel on days 5, 7, 9, and 13. There was no significant difference in the wound healing percentage between wounds treated with CMS only and the control group. These results indicated that CMS@2%CuO nanocomposite hydrogel accelerates wound healing. The control group had 31%, 46%, and 79% wound healing on days 7, 9, and 13, respectively, whereas the CMS@2%CuO nanocomposite hydrogel group had 76%, 84%, and 94% wound healing for the respective time periods. Wound length was measured on days 0, 3, 5, 7, 9, and 13 after the creation of the wound. Wound length was significantly reduced in the CMS@2%CuO hydrogel group on day 13, when compared with the CMS group or the control group (Figure 7d). The findings indicate that wound healing in rats was accelerated with the use of the CMS@2%CuO nanocomposite hydrogel as a wound dressing. On day 13, almost complete healing of the wound (94%) was observed in CMS@2%CuO hydrogel-treated wounds. In contrast, wound healing was only 82% and 79% for CMS and control groups, respectively. Improvement in wound healing is likely attributed to the release of Cu(II) ion, an essential trace element with potent biocidal properties that promotes skin generation and angiogenesis [56,57]. Copper is believed to be involved in many processes related to wound healing. For example, angiogenesis is increased in the presence of copper (II) ions via induction of vascular endothelial growth factor production, enhancement of fibrinogen, and collagen stabilization, as well as an increase in the activity of the copper-dependent enzymes involved in matrix remodeling, cell proliferation and re-epithelization [57,58,59]. There is evidence that copper deficiency impairs the wound healing process [60]. Copper oxide nanoparticles produced by green synthesis have been reported to inhibit the growth of pathogenic bacteria on excision wounds and accelerated wound healing in rats [24]. The present findings are in agreement with the literature that copper oxide nanoparticles accelerate wound healing [24,61].

## 3. Discussion

Wound care is essential in the elderly, as well as in diabetic and immunocompromised patients. When a wound is formed, microbes grow rapidly at the wound site. Depending on the type of the wound and its location, as well as how the wound is taken care of, the body initiates the wound healing process. The healing process may be interrupted by both external and internal agents. Four overlapping healing phases are involved in wound healing: hemostasis, inflammation, proliferation, and remodeling [62]. Microbial infections and ROS are important perturbing factors in the wound healing process and can prolong each healing phase, consequently resulting in structurally and functionally unsatisfactory results [63].

**Figure 8:**
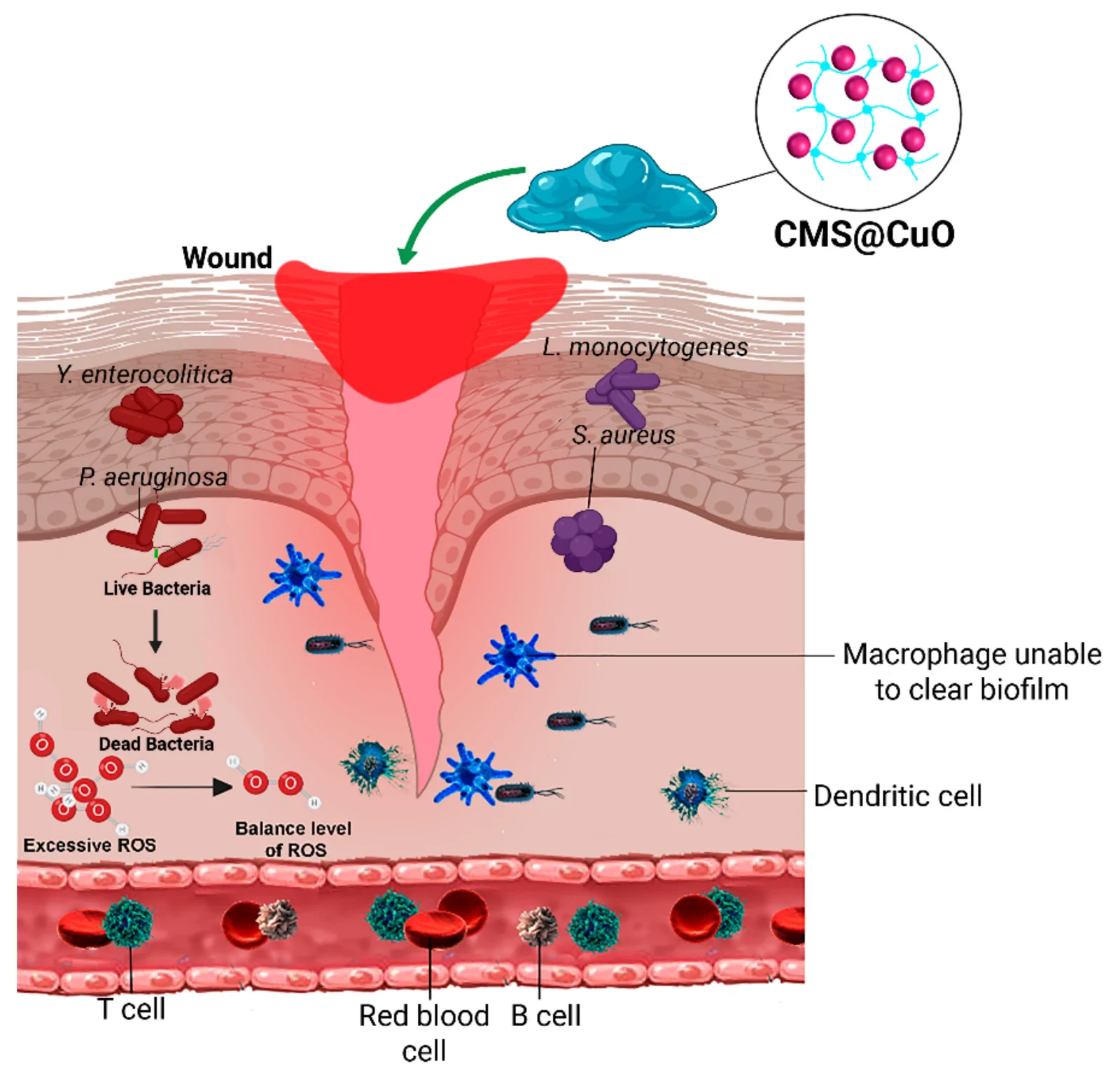
Schematic of the application of a nanocomposite hydrogel with antibacterial and antioxidant properties to promote wound healing.

In healthy skin, the microbiota within this skin barrier is fairly stable. They through producing antimicrobial compounds and occupying various receptors on host cells limit invasion of pathogenic microbes. Although, commensal skin microbiota that exists beyond a critical threshold and microbial pathogens such as *S. aureus*, methicillin-resistant *S. aureus*, *P. aeruginosa,* and *E. coli* are among microorganisms which can cause infection in the wound site and adversely affect the initial phases of wound healing as well as healing of chronic wounds. When these microbial cells passed through the injured skin various innate and acquired immune cells such as dermal dendritic cells, macrophages, mast cells, T and B cells via various strategies try to remove pathogens. Agents with wound healing activity are used to accelerate healing and minimize wound-related complications. Although taking systemic antibiotics helps the body prevent microbial infections [64], the application of a locally-applied antimicrobial dressing on a wound is more desirable. Firstly, orally-administered antibiotics have to be metabolized in the gastrointestinal tract. Thus, a high concentration of antibiotics has to be taken for it to reach the wound site. Secondly, localized administration of antibiotics reduces microbial resistance to these drugs, which is a growing concern [43]. Pomades, gels, and ointments can kill the bacteria that multiplied at the wound site and may reduce the duration of treatment [65]. These types of materials are particularly useful in patients with diabetes, hepatitis, and acquired immune deficiency syndrome who have defective immune systems [66,67]. In the present study, the nanocomposite hydrogels investigated possess broad-spectrum antimicrobial activity and significant antioxidant ability. Results from the present study showed that the CMS and CMS@CuO hydrogels were able to kill eight species of Gram-positive and Gram-negative bacteria that are pathogenic to humans. When a wound is in the inflammatory phase, a large amount of ROS such as superoxide (O_2_), peroxynitrite (ONOO^−^), and hydroxyl radicals (OH) are generated that damage the body’s proteins and DNAs [6]. The use of antioxidant materials orally or topically can control ROS and prevent oxidative stress. Once the level of the antioxidant molecules is inadequate to protect the cells and tissues from excessive oxidative stress, delay in wound healing is inevitable [6,12]. The CMS@CuO hydrogels investigated in the present study have antioxidant properties that are higher than 75%. The potent antioxidant activity enables the elimination of free radicals from the wound site. The in vivo data generated from the present study demonstrate that the application of these hydrogels as dressings in the wound site accelerates wound healing. These attributes synergistically support wound healing. Accordingly, the tested null hypothesis that “there is no difference in the antioxidant activity, antimicrobial activity, and in vivo wound healing potential between hydrogels prepared from CMS incorporating CuO nanoparticles (CMS@CuO) and those prepared with CMS only” has to be rejected. Figure 8 schematically illustrates the application of the nanocomposite hydrogel with antibacterial and antioxidant activity to promote wound healing.

## 4. Materials and Methods

### 4.1. Materials

Corn starch (practical grade with 24.35% amylose and 13.27% moisture content) was acquired from Kimia Eksir company (Tehran, Iran). Sodium hydroxide, isopropanol, monochloroacetic acid (MCA), methanol, citric acid (CA), and 2,2-diphenyl-1-picrylhydrazyl (DPPH·) were purchased from Merck KGaA (Darmstadt, Germany) and used without purification. Spherical CuO nanoparticles, each with a mean diameter of ~30 nm, were acquired from Neutrino Company (Tehran, Iran). All Gram-negative and Gram-positive bacteria were purchased from Persian Type Culture Collection (PTCC; Tehran, Iran) and the American type culture collection (ATCC; Manassas, VA, USA).

### 4.2. Synthesis of Sodium Carboxymethyl Starch (CMS)

**Figure 9:**
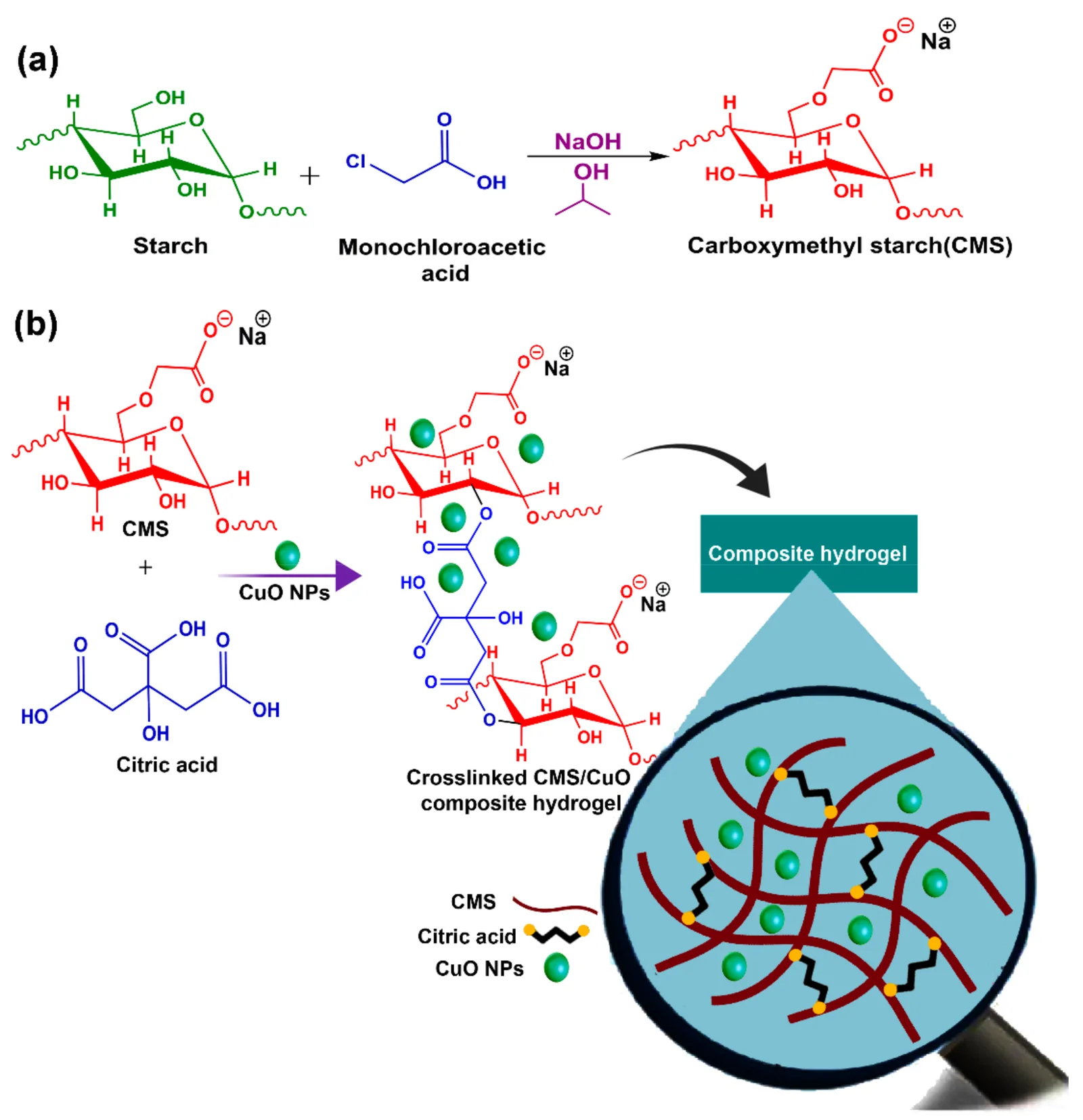
Schematic of the preparation of (**a**) sodium carboxymethyl starch (CMS) and (**b**) nanocomposite hydrogels that consists of CuO nanoparticles (NPs) dispersed within the citric acid crosslinked CMS polymer matrix.

Sodium carboxymethyl starch (CMS) was synthesized using a previously-reported method with minor modifications [16] (Figure 9a). Corn starch (1 g) was dispersed and stirred in 15 mL isopropanol for 15 min. Sodium hydroxide (1.2 g) was slowly added to the solution, with continuous stirring at 40 °C for 1 h. This was followed by the addition of 1.36 g of monochloroacetic acid to the solution, with continuous stirring for another 1 h. A white precipitate of CMS was subsequently obtained. The precipitate was centrifuged, washed several times with 80% methanol solution, and dried at 40 °C for 24 h. The degree of substitution (DS = 0.87) of the CMS was calculated by the direct titration technique [68].

### 4.3. Fabrication of CMS@CuO Nanocomposite Hydrogels

Nanocomposite hydrogels consisting of a sodium CMS matrix and CuO nanoparticles (CMS@CuO) were fabricated using the solution-casting method (Figure 9b). For small-scale synthesis, 1 g of CMS powder was mixed with 50 mL of distilled water and stirred at room temperature until completely dissolved. The CuO nanoparticles (2 wt.% and 4 wt.%) were added to the solution and sonicated at ambient temperature for 10 min. The citric acid (15 wt.% to the CMS) was used as the crosslinking agent and gradually added to the reaction vessel. The solution was kept at ambient temperature for 30 min. The mixture was poured into plastic plates and dried at 30 °C. In the present work, CMS@CuO nanocomposite hydrogels with 2 wt.% or 4 wt.% CuO nanoparticles were fabricated. A control hydrogel was also prepared using crosslinked CMS without incorporation of CuO nanoparticles.

### 4.4. Characterization

The prepared nanocomposite hydrogels were examined using Fourier transform infrared spectroscopy (FTIR) (Equinox 55, Bruker Optik GmbH, Leipzig, Germany) in the wavenumber range of 4000–400 cm^−1^. A field emission scanning electron microscope coupled with energy dispersive X-ray analysis (FESEM/EDS) (MIRA 3-XMU, Tescan, Kohoutovice, Czech Republic) was used for evaluating the surface morphology of the synthesized materials. The crystallinity of materials was examined using X-ray diffraction (XRD) (D8 Advance X-ray diffractometer, Bruker Optik GmbH, Leipzig, Germany). Thermogravimetric analysis (TG 209F3, NETZSCH, Selb, Germany) was used to investigate the thermal stability of the materials. Ultraviolet-visible light absorption spectroscopy (Cecil 5000 series UV–vis spectrometer (Cecil Instruments Ltd., Cambridge, UK) was used for evaluating the antioxidant activities of materials.

### 4.5. Swelling Study

Fluid absorbance was evaluated by first weighing the dried hydrogel specimens at 40 °C (W_0_, primary mass). The dried specimens were placed in glass vessels with 10 mL distilled water at room temperature for 1 h. The hydrated hydrogel specimens were removed from the glass vessels, gently wiped with filter paper to eliminate excess water, and re-weighed (W_s_, swollen mass). Weight values were used to calculate the degree of swelling (SD, in %) using the following Equation (1) [28].

SD(%) = ((W_s_ − W_0_)/W_0_) × 100% (1)

### 4.6. Antioxidant Study

The hydrogel specimens were placed in beakers containing distilled water for 24 h. One milliliter of the hydrogel solution was mixed with 2 mL of methanolic DPPH· solution (25 μmol/L) and kept in a dark place for 30 min. Solution absorbance was measured at 517 nm using the aforementioned UV–vis spectrophotometer. The antioxidant activity of pristine CMS and the CuO nanoparticles were also measured for comparison. Inhibition of DPPH (in %) was measured using the following Equation (2) [49]:

DPPH inhibition (%) = (A_b_ − A_s_/A_b_) × 100 (2)

where A_b_ is the methanolic DPPH absorption (blank) and A_s_ is the absorption of the sample solution.

### 4.7. Antibacterial Study

The Kirby–Bauer test (disk-diffusion method) was used to compare the antibacterial efficacy of CuO nanoparticles, CMS, CMS@2%CuO, and CMS@4%CuO against bacterial growth. Several Gram-positive bacteria (*Listeria monocytogenes*, *Enterococcus faecalis*, *Staphylococcus aureus* and methicillin-resistant *Staphylococcus aureus*) and Gram-negative bacteria (*Salmonella enterica*, *Pseudomonas aeruginosa*, *Escherichia coli*, and *Yersinia enterocolitica*) were used to test the antibacterial efficacy of the hydrogel nanocomposites. The Lyophilized bacteria were revived in their corresponding medium at 37 °C for 24 h. After incubation, 10 µL of each microbial suspension was added to culture medium-containing agar and incubated at 37 °C for 24 h. For determination of antimicrobial activity, 4–6 identical colonies of each microbial species were inoculated into tubes containing Muller Hinton broth or tryptic soy broth. The inoculated media was incubated at 37 °C for 4 h. At the end of the incubation period, the optical density of each medium was read at 625 nm. The absorbance of media was adjusted to 0.08–0.1 with a final cell density of 1.5 × 10^8^ colony forming units (CFU)/mL. These microbial suspensions were spread on Muller Hinton agar or tryptic soy agar using a sterile cotton swab. Sterile CuO NPs, CMS, CMS@2%CuO, and CMS@4%CuO (8 mm diameter) were inoculated on agar-containing Petri plates and incubated at 4 °C to enable diffusion of compounds derived from the tested specimens into the medium. The plates were incubated at 37 °C for 24 h prior to measuring the diameter of the inhibition zones on the surface of the agar. Each test was performed in triplicate and the results were reported as means ± standard deviations.

### 4.8. In Vitro Cell Cytotoxicity Study

The cytotoxicity of the fabricated nanocomposite hydrogels and CMS on human dermal fibroblast (HDF-with NCBIcode of C645; Cell Bank of Pasteur Institute of Iran, Tehran, Iran) was evaluated using the MTT (3-(4,5-dimethylthiazol-2-yl)-2,5-diphenyltetrazolium bromide) assay after incubation for 24, 48 and 72 h. The stock solution of the specimens was prepared in dimethyl sulfoxide (DMSO), and 25, 50, 100, 200, 600, 800, and 1600 µg mL^−1^ of the specimens were prepared using Dulbecco’s Modified Eagle Medium. The cytotoxicity of DMSO, the solvent of nanocomposite hydrogels and CMS, was also determined. Untreated cells were used as the control. The viability of the cells exposed to the samples was calculated using the following Equation (3):

(3) $$\mathrm{Viability}\%\;=\;\frac{\mathrm{Absorbance}\;\mathrm{in}\;\mathrm{the}\;\mathrm{Treatment}\;\mathrm{Group}}{\mathrm{Absorbance}\;\mathrm{in}\;\mathrm{Control}\;\mathrm{Group}}\times 100$$

### 4.9. In Vivo Wound Healing Study

The efficacy of the hydrogels on wound healing was evaluated using a small animal model. Male Wistar rats were randomly divided into three groups: control, CMS, and CMS@2%CuO. The rats were anesthetized with an intraperitoneal injection of 10 mg/kg Rampan (Bayer, Leverkusen, Germany) and 100 mg/kg ketamine hydrochloride (Gedeon Richter, Budapest, Hungary). Hair from the back of each rat was shaved and cleaned. A full-thickness incision (35 mm in length) was created at a distance of 1.5 cm on the right of the dorsal midline of each rat (Figure 7). Wounds in the CMS and CMS@2%CuO groups were treated with CMS and CMS@2%CuO hydrogels, respectively. The results were compared to the untreated control group. Wound length and wound surface area were used for healing evaluation. For surface area measurement, each rat was anesthetized with ether inhalation. A trace of each wound surface was made with tracing paper affixed to a millimeter paper. Squares located inside the trace were counted. The surface area was measured on 0 (surgery day), 3, 5, 7, 9, and 13 days after surgery. The percentage of wound healing percentage was determined using the following Equation (4) [69].

(4) $$\mathrm{Wound}\;\mathrm{healing}(\%)\;=\;\frac{\left[Wound\;surface\;area\;on\;\left(0\right)days\right]-\;\left[Wound\;surface\;area\;on\;\left(x\right)days\right]}{\left[Wound\;surface\;area\;on\;\left(0\right)days\right]}\times 100$$

### 4.10. Statistical Analysis

Data were expressed as means and standard deviations. For each experiment that required statistical analysis, data in the respective groups were tested for their normality (Shapiro-Wilk test) and equal variance assumptions (modified Levene test). If those assumptions appeared to have been violated, the data were non-linearly transformed to satisfy those assumptions prior to the use of parametric statistical methods. For each data set, results derived from the associated groups were analyzed using a one-factor analysis of variance. Post-hoc pairwise comparisons were performed using the Tukey test. For all tests, statistical significance was present at α = 0.05.

## 5. Conclusions

In the present work, CMS and CMS@CuO hydrogels were fabricated by solution-casting and investigated as potential wound healing. The hydrogels were characterized using different methods to determine their physicochemical properties. The CMS-based hydrogels containing CuO nanoparticles exhibited a high swelling degree (above 80%) when crosslinked with an optimized amount of citric acid (15 wt. %). The CMS@CuO nanocomposite hydrogels showed good antibacterial activity against eight types of Gram-negative and -positive bacteria that are pathogenic to the human body. Maximum antioxidant activity was observed in hydrogels containing 4 wt.% CuO nanoparticles. Low cytotoxicity against human fibroblasts was observed in the nanocomposite hydrogel that contains 2 wt.% CuO nanoparticles. Evaluation of in vivo wound healing using a rat model indicates that skin wounds treated with the CMS@2%CuO nanocomposite hydrogel healed faster than wounds treated with the pure CMS hydrogel. Incorporation of CuO nanoparticles into CMS improved antibacterial and antioxidant activities. The CMS@CuO nanocomposite hydrogel has the potential to be used as a skin wound dressing in tissue regeneration.

## Supplementary Materials

Supplementary data are available online at https://www.mdpi.com/1422-0067/22/5/2531/s1, Figure S1: FTIR spectrum (a), XRD pattern (b), EDS spectrum (c), and TGA curve (d) of the CMS@4%CuO nanocomposite hydrogel, Table S1: UV-vis data of antioxidant activities of CMS, CuO nanoparticles, CMS@2%CuO, and CMS@4%CuO nanocomposite hydrogels in methanolic DPPH· solution. Ab, the absorbance of DPPH solution at 517 nm; As, the absorbance of each sample in DPPH solution at 517 nm.
