# Correction: Abdollahi et al. Bioactive Carboxymethyl Starch-Based Hydrogels Decorated with CuO Nanoparticles: Antioxidant and Antimicrobial Properties and Accelerated Wound Healing In Vivo. *Int. J. Mol. Sci.* 2021, *22*, 2531

**DOI:** 10.3390/ijms27135896

**Published:** 2026-06-30

**Authors:** Zahra Abdollahi, Ehsan Nazarzadeh Zare, Fatemeh Salimi, Iran Goudarzi, Franklin R. Tay, Pooyan Makvandi

**Affiliations:** 1 School of Chemistry, Damghan University, Damghan 36716-41167, Iran; zahra.yalda.abdollahi@gmail.com; 2 School of Biology, Damghan University, Damghan 36716-41167, Iran; f.salimi@du.ac.ir (F.S.); irangoudarzi@du.ac.ir (I.G.); 3 The Graduate School, Augusta University, Augusta, GA 30912, USA; tayfranklin7@gmail.com; 4 Istituto Italiano di Tecnologia, Centre for Materials Interface, Viale Rinaldo Piaggio 34, 56025 Pontedera, Pisa, Italy

## Error in Figure

In the original publication [1], there was a mistake in Figure 7 as published. The photograph of the wound on day 3 for the CMS material was similar to that of the control sample. The authors inadvertently made an error in this image and apologize; the corrected photograph has now been provided. The corrected Figure 7 updated with the corrected panel for CMS day 3 appears below.

## Missing Funding and Ethical Approval

In the original publication, the Funding statement and Institutional Review Board Statement were incomplete. The corrected statements appear below.

**Funding:** This research received institutional support from Damghan University for materials and infrastructure. No external grant funding was utilized.
**Institutional Review Board Statement:** Ethical approval for this study was formally issued in Persian by the Research Ethics Committee of Damghan University prior to the commencement of the animal experiments, as required. The original approval document bears the official institutional seal, approval code REC-DU-2020-334, 4 January 2020, confirming that ethical clearance was obtained before the study began.

The authors state that the scientific conclusions are unaffected. This correction was approved by the Academic Editor. The original publication has also been updated.

## Figures and Tables

**Figure 7:**
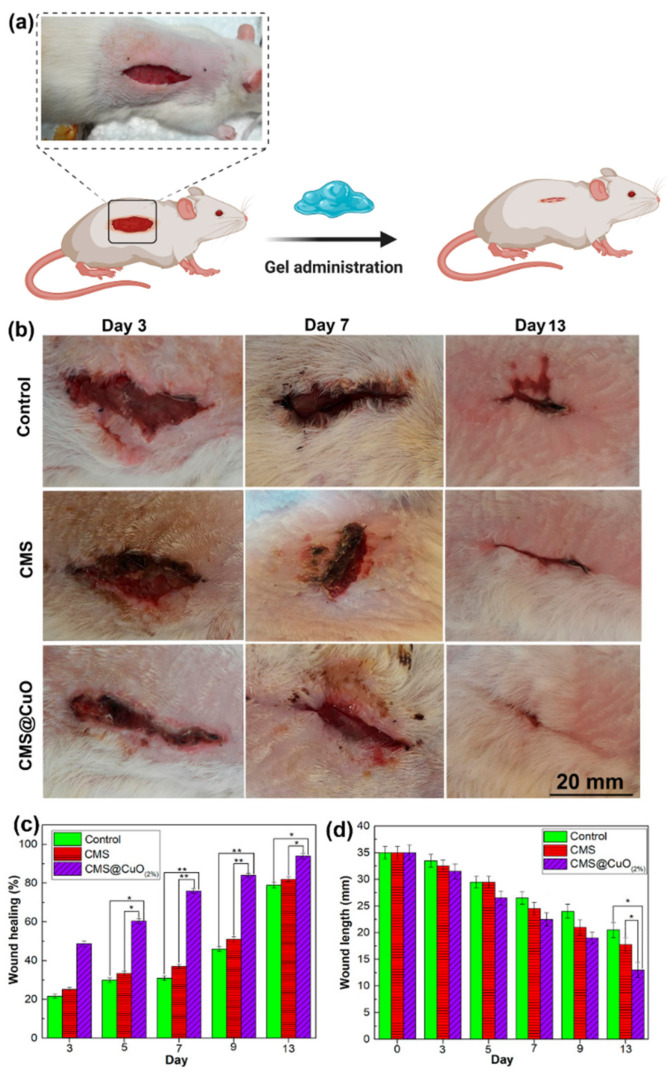
Macroscopic photographs of the wounds treated with the control, CMS, and the CMS@2%CuO nanocomposite hydrogel specimens at different time periods (**a**,**b**) and histograms of the percentage of wound healing (**c**) and wound length (**d**). For each chart, columns labeled with an asterisk (*^,^**) are significantly different from the control (*p* < 0.05 and *p* < 0.01).
