# Supplementary material for: Bioactive Carboxymethyl Starch-Based Hydrogels Decorated with CuO Nanoparticles: Antioxidant and Antimicrobial Properties and Accelerated Wound Healing In Vivo
Source: Int J Mol Sci. 2021 Mar 3;22(5):2531. doi: 10.3390/ijms22052531 (PMC7959477; doi:10.3390/ijms22052531)
Supplement: Supplementary file 1 [file ijms-22-02531-s001.pdf]

Supplementary Material

Bioactive Carboxymethyl Starch-Based Hydrogels Decorated with CuO Nanoparticles: Antioxidant and Antimicrobial Properties and Accelerated Wound Healing In Vivo

Zahra Abdollahi [1], Ehsan Nazarzadeh Zare [1,*], Fatemeh Salimi [2], Iran Goudarzi [2], Franklin R. Tay [3], and Pooyan Makvandi [4,*]

1 School of Chemistry, Damghan University, Damghan 36716-41167, Iran; zahra.yalda.abdollahi@gmail.com
2 School of Biology, Damghan University, Damghan, 36716-41167, Iran; f.salimi@du.ac.ir (F.S.); irangoudarzi@du.ac.ir (I.G.)
3 The Graduate School, Augusta University, Augusta, GA 30912, USA; tayfranklin7@gmail.com
4 Istituto Italiano di Tecnologia, Centre for Materials Interface, viale Rinaldo Piaggio 34, 56025 Pontedera, Pisa, Italy
* Correspondence: ehsan.nazarzadehzare@gmail.com; e.nazarzadeh@du.ac.ir; Tel.: +982335220095 (E.N.Z.); pooyanmakvandi@gmail.com (P.M.)

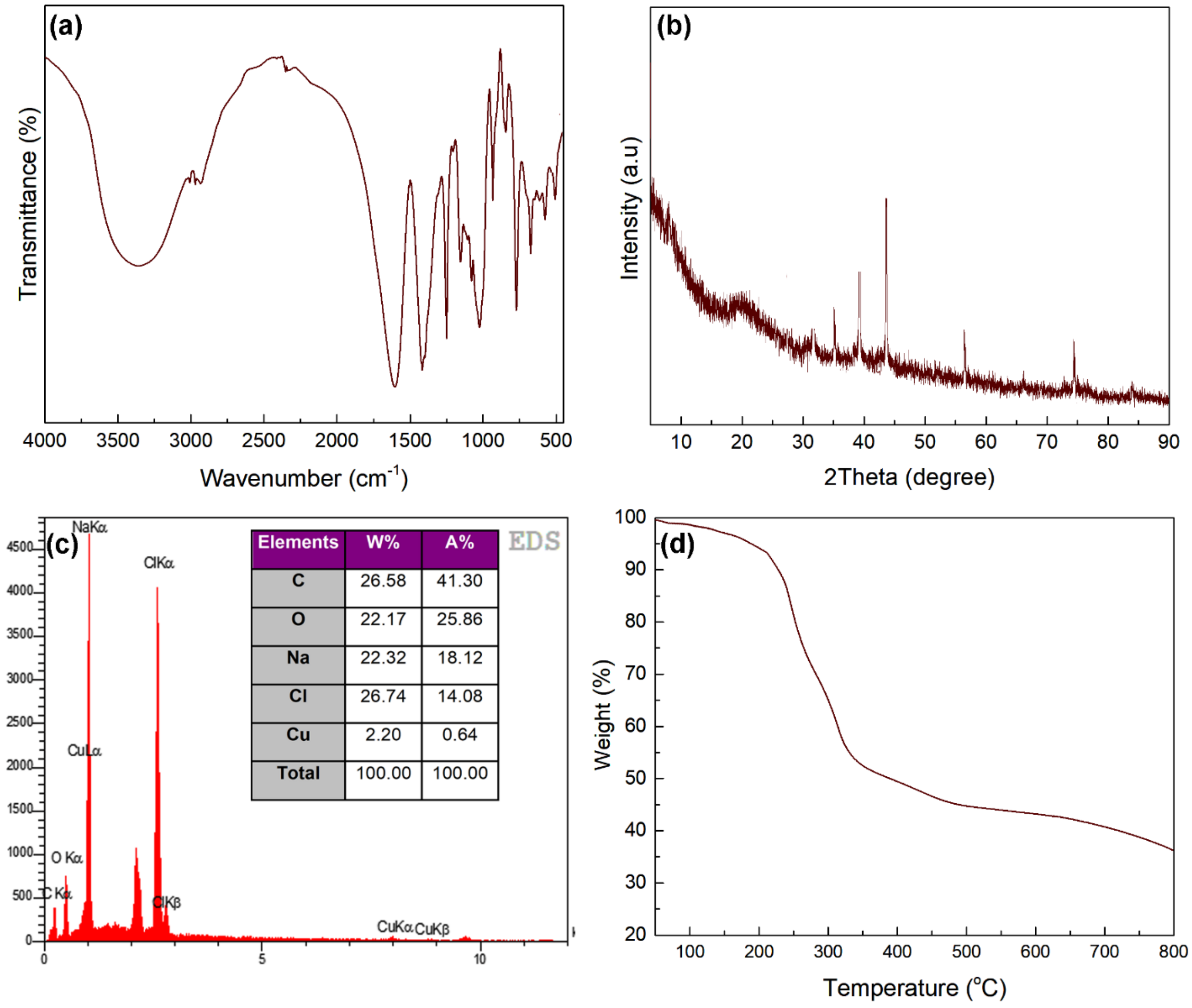

Elements	W%	A%
C	26.58	41.30
O	22.17	25.86
Na	22.32	18.12
Cl	26.74	14.08
Cu	2.20	0.64
Total	100.00	100.00

Figure S1. FTIR spectrum (a), XRD pattern (**b**), EDS spectrum (**c**), and TGA curve (**d**) of the CMS@4%CuO nanocomposite hydrogel.

Table S1. UV-vis data of antioxidant activities of CMS, CuO nanoparticles, CMS@2%CuO, and CMS@4%CuO nanocomposite hydrogels in methanolic DPPH· solution. Ab, the absorbance of DPPH solution at 517 nm; As, the absorbance of each sample in DPPH solution at 517 nm.

**Samples**	**A_b**	**A_s**	**DPPH %**
CMS	0.450	0.283	37
CuO nanoparticles	0.450	0.112	75
CMS@2%CuO	0.450	0.090	80
CMS@4%CuO	0.450	0.081	82
